# The Lymphatic–Bone Axis in Cancer Metastasis

**DOI:** 10.3390/cancers18060892

**Published:** 2026-03-10

**Authors:** Ahlim Lee, James Rhee, Rajeev Malhotra, Jang Hee Han, Kangsan Roh

**Affiliations:** 1 Department of Urology, Seoul National University Hospital, Seoul National University College of Medicine, Seoul 03080, Republic of Korea; megimegi7970@skku.edu; 2 Cardiovascular Research Center, Massachusetts General Hospital, Harvard Medical School, Boston, MA 02114, USA; 3 Division of Cardiology, Department of Medicine, Massachusetts General Hospital, Harvard Medical School, Boston, MA 02114, USA; 4 Department of Anesthesia, Critical Care, and Pain Medicine, Massachusetts General Hospital, Boston, MA 02114, USA; jrhee@mgb.org

**Keywords:** bone metastasis, lymphatic system, lymph node metastasis, high endothelial venules, osteomimicry, lymphatic–bone axis

## Abstract

Bone metastasis is a devastating complication of advanced osteotropic malignancies, notably breast, prostate, lung carcinomas, and malignant melanoma, and remains a primary driver of mortality. Historical paradigms have conceptualized skeletal dissemination almost exclusively as a hematogenous process wherein circulating tumor cells colonize receptive bone marrow niches. However, this model fails to reconcile why lymph node metastasis consistently serves as a potent predictor of bone involvement even though therapeutic lymphadenectomy rarely prevents distant spread. This discordance suggests that lymph nodes function not merely as passive reservoirs but as active ‘evolutionary gateways’ that sculpt bone-tropic metastatic clones. In this review, we introduce the Lymphatic–Bone Axis, a framework integrating lymphatic biology into models of bone metastasis. We synthesize emerging evidence elucidating how the lymph node microenvironment primes tumor cells through CCR7-CXCR4 switching, induction of osteomimicry programs, and metabolic reprogramming that favors survival within the bone marrow. We also discuss preclinical data demonstrating direct intranodal intravasation via high endothelial venules (HEVs), providing a rapid route into the systemic circulation that bypasses the thoracic duct. Beyond consolidating current knowledge, we outline a research agenda for dissecting this axis, including longitudinal single-cell transcriptomic mapping and functional assessments of lymph node-derived tumor cells. Finally, we consider translational implications, highlighting why bone-targeted agents alone may prove insufficient once cells are conditioned within lymphatic niches. By mechanistically linking lymphatic priming to skeletal colonization, this review informs the rational design of multimodal therapeutic approaches that jointly target lymphatic transit and the bone microenvironment.

## Simple Summary

Bone metastasis is a fatal complication of cancer that causes fractures and severe pain. Historically, it was thought that cancer cells reached bones solely through the bloodstream. However, patients with lymph node metastases are at high risk for bone lesions, yet surgical removal of these nodes often fails to prevent further spread. This suggests that lymph nodes function as more than passive filters; they may actively modify cancer cells to survive in the bone environment. This review introduces the Lymphatic–Bone Axis, describing how the lymph node environment alters cancer cells to express specific bone-homing proteins and acquire bone-like traits. We also discuss evidence that cancer cells can enter the bloodstream directly from lymph nodes through specialized blood vessels, bypassing standard routes. These insights suggest that new treatments should target these adaptive processes within the lymphatic system before cancer cells colonize bone.

## 1. Introduction

Bone metastasis is a major clinical challenge in advanced cancers [1]. For patients with advanced prostate or breast cancer, this complication is particularly frequent, affecting over 70% of individuals [2,3,4]. The development of skeletal lesions often leads to severe morbidity, including pathological fractures, spinal cord compression, and debilitating pain [5,6]. Current treatments, such as bone-resorbing agents, primarily manage established lesions rather than prevent their initial formation [7,8]. While less prevalent than in breast or prostate carcinomas, bone involvement in advanced melanoma is also a significant clinical entity associated with poor prognosis. This limitation underscores the need for a more complete understanding of the metastatic process [1,7].

Historically, cancer dissemination has been explained by the hematogenous model, which posits that cancer cells metastasize by entering the bloodstream to colonize distant organs [9,10]. Within this framework, the bone’s highly vascularized marrow is viewed as a receptive site for these circulating tumor cells [11].

However, a singular focus on this blood-borne route fails to adequately explain robust clinical data suggesting that lymph node metastasis (LNM) is a powerful predictor for subsequent bone metastasis [12]. This link is evident across several key cancer types. In prostate cancer, for which bone is the most common site of distant spread, lymph node status has been identified as a significant independent predictor of bone metastasis [13,14]. This clinical correlation is also exceptionally strong in breast cancer and melanoma [15]. For these patients, a negative sentinel lymph node (SLN) biopsy correlates with a significantly lower incidence of distant metastasis, with recurrence rates reported as low as 5–10% [16]. This strongly suggests that for these cancers, the SLN may serve as the major gateway for systemic dissemination [17]. This consistent clinical link where the status of the regional lymph node strongly predicts the development of distant skeletal disease motivates a deeper investigation into the lymphatic system’s functional role in disseminating cells to bone, particularly as a direct anatomical pathway is not obvious [18,19,20].

This review will overview current models of bone metastasis and interactions within the bone microenvironment, before integrating the underappreciated role of the lymphatic system to provide a holistic perspective on this lethal stage of cancer.

## 2. Current Perspectives on Bone Metastasis

### 2.1. The Hematogenous Metastatic Cascade

**Figure 1:**
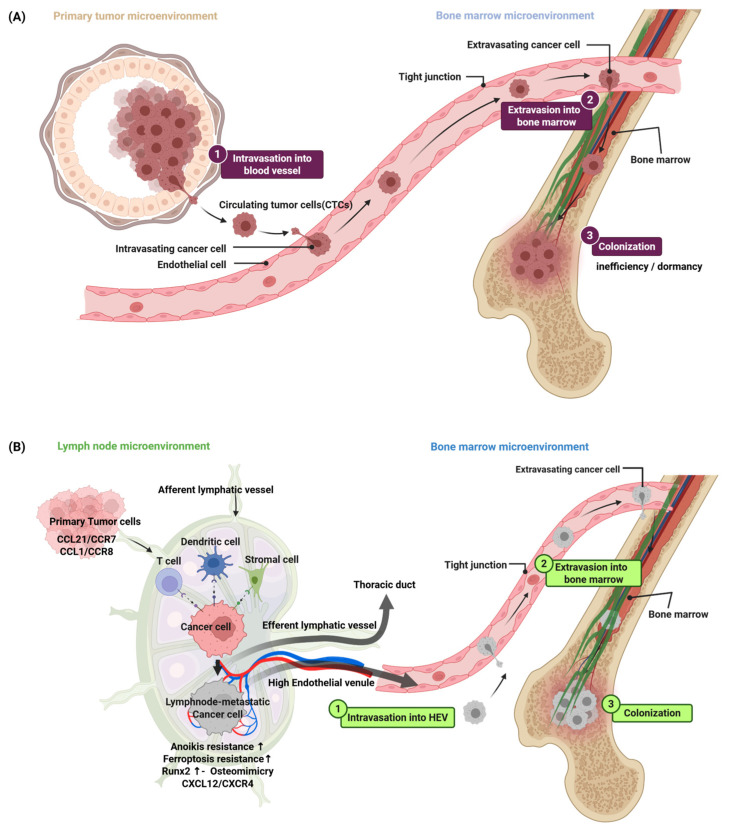
Contrasting Models of Metastatic Dissemination to the Bone Marrow. (**A**) The classical hematogenous metastasis model. Cancer cells from the primary tumor microenvironment intravasate directly into the bloodstream. These circulating tumor cells (CTCs) disseminate systemically, adhere to the bone marrow vasculature, and extravasate to colonize the bone, a process often characterized by a period of inefficiency or dormancy. (**B**) The proposed lymphatic–hematogenous hybrid model. Cancer cells from the primary tumor exploit chemokine gradients such as CCL21–CCR7 and CCL1–CCR8 to invade afferent lymphatic vessels and migrate to the draining lymph node. Within the lymph node microenvironment, tumor cells interact with stromal and immune components such as T cells and dendritic cells, undergoing a ‘priming’ process. This selection and adaptation phase is hypothesized to enrich for cells with specific acquired traits essential for distant colonization, including resistance to anoikis and ferroptosis, upregulation of the osteomimetic transcription factor RUNX2, and activation of the CXCL12–CXCR4 axis, a shared homing pathway for both lymph nodes and bone marrow. These ‘primed’ lymph node-metastatic cells subsequently bypass the thoracic duct by intravasating directly into the intranodal blood vasculature via high endothelial venules (HEVs). Upon entering the systemic circulation, they follow conventional hematogenous routes to efficiently colonize the bone marrow microenvironment, potentially bypassing the dormancy phase observed in the classical model. Arrows indicate the direction of cancer cell dissemination. Color coding highlights distinct dissemination routes, including hematogenous circulation (pink) and lymphatic pathways (green).

The conventional model of bone metastasis describes a multi-step sequence primarily driven by hematogenous dissemination (Figure 1A) [21]. The process is thought to begin with cancer cells detaching from the primary tumor and penetrating the walls of intratumoral or nearby blood vessels, a step known as intravasation [22]. The survival and destination of these circulating tumor cells (CTCs) are not random [23,24]. Evidence suggests that bone-tropic metastatic seeds may be pre-selected within the primary tumor [25]. For instance, cancer cells with high Src kinase activity [26] or expression of specific chemokine receptors like CXCR4 [27] may be inherently primed to survive and home to the bone microenvironment, which is rich in their corresponding ligands, such as CXCL12 [28].

Upon reaching the skeleton, these CTCs adhere to the endothelium of bone marrow vessels [29]. This is followed by extravasation, a process of migration from the vessel into the marrow stroma [30]. The bone marrow vasculature is heterogeneous [31], containing distinct capillary types such as type H and type L vessels [32]. The subsequent tumor cell adhesion and extravasation are not random events but are thought to be mediated by highly specific molecular interactions [33,34]. Key among these are the binding of endothelial E-selectin [35] and the interaction of integrins, such as VLA-4(α_4_β_1_) [36], on tumor cells with their ligands, like VCAM-1 [37], on marrow stromal and endothelial cells [38]. This homing process is further directed by potent chemokine gradients, most notably the previously mentioned CXCL12-CXCR4 axis [39].

Once inside the marrow, cells may colonize distinct niches that determine their fate [40]. The perivascular niche, for example, has been associated with a state of cellular dormancy [41], where cells can remain quiescent for extended periods that can last from years to even decades [42]. This quiescent state is thought to be actively induced by signals from the niche [43], such as thrombospondin 1 (TSP1) secreted by endothelial cells or TGFβ2 from perivascular cells [44]. In contrast, the osteogenic niche, located near osteoblasts on the endosteal surface, is linked to reactivation and proliferation [40]. This outgrowth is promoted by direct cell-to-cell contact, including the formation of heterotypic adherens and gap junctions, as well as signaling through pathways like Notch [45]. These interactions are dynamic and can vary significantly between different metastatic lesions [33]. These biological features collectively align with observed clinical patterns of dissemination. Clinically, metastases show a strong preference for the axial skeleton such as the spine, pelvis, and ribs, which is highly vascularized and rich in hematopoietic marrow [4,33]. This distribution is often attributed to the vertebral-venous (Batson’s) plexus, a low-pressure valveless system that allows venous blood from the breast and pelvis to bypass the lungs and gain access to the spine [46]. However, this model alone cannot explain why lymph node status is such a powerful predictor of bone metastasis (Table 1), suggesting that additional, lymphatic-dependent mechanisms may be operative.

### 2.2. The Bone Microenvironment and Pre-Metastatic Niche

The bone is considered a uniquely receptive site for metastasis due to its complex microenvironment [61,62]. The intrinsic properties of the BME include active hematopoiesis and continuous remodeling a balanced process of bone resorption by osteoclasts and formation by osteoblasts [63,64]. The bone matrix also contains a rich depot of growth factors [65]. During remodeling, matrix-embedded factors such as TGF-β, IGFs, Bone Morphogenetic Proteins (BMPs), Fibroblast Growth Factors (FGFs), and Platelet-Derived Growth Factors (PDGFs) can be liberated. While in vitro and animal models robustly suggest these factors encourage the growth of colonized tumor cells [62,66,67], whether the local physiological concentrations of these factors within the in vivo human bone microenvironment are strictly sufficient to independently drive metastasis remains a challenging question and a subject of ongoing investigation.

Beyond these inherent features, the BME is not a passive recipient [61]. Evidence suggests it can be actively conditioned by the primary tumor before metastatic cells arrive [68]. This concept is known as pre-metastatic niche (PMN) formation [68,69].

Bone-specific conditioning mechanisms have been most extensively studied in osteolytic metastasis models, where tumor-derived factors such as RSPO2 promote osteoclast-mediated pre-metastatic niche formation through LGR4–RANKL signaling [70]. This process is initiated by tumor-secreted factors and represents a bone-specialized mechanism, distinct from soft-tissue pre-metastatic niche formation, which typically relies on the recruitment of bone marrow–derived cells (BMDCs) [39]. For instance, in breast cancer models, hypoxic primary tumors secrete the enzyme lysyl oxidase (LOX) [71], which is reported as essential for the formation of osteolytic PMN lesions [72]. Other systemically secreted factors like PTHrP may also “prime” the bone by activating osteoblasts to release RANKL [73], thereby inducing osteoclast differentiation even before cancer cells are present [74]. For osteoblastic metastases, commonly seen in prostate cancer [75], less is known about PMN formation, but candidate factors secreted by primary tumors include WNT proteins [76], BMPs [77], and endothelin-1 [78], which may promote an osteoblast-receptive environment.

This organ-specific preparation is a key feature of the PMN, distinguishing it from other sites. Studies show that tumor-derived exosomes [79], for example, can be directed to specific organs based on the integrins they express on their surface. Exosomal integrins like α_6_β_4_ promote homing to the lung PMN [80], whereas α_ν_β_5_ targets exosomes to the liver PMN [81]. This highlights that the pre-metastatic conditioning is a highly targeted process. The successful establishment of metastasis may therefore depend on both the properties of the cancer cell and this acquired organ-specific receptivity of the BME.

### 2.3. Lesion Heterogeneity and Cancer-Specific Patterns

The interaction between colonized cancer cells and the BME results in distinct lesion types [62,82]. Osteolytic lesions, common in breast cancer, are characterized by bone destruction [83]. This process appears to be driven by tumor-secreted factors that enhance osteoclast activity, creating a self-reinforcing vicious cycle [84]. Conversely, osteoblastic (or sclerotic) lesions, predominant in prostate cancer, involve excessive and disorganized bone formation stimulated by tumor-derived factors [84,85]. Notably, even in cancers that preferentially cause osteolytic lesions (like breast cancer), osteoblastic lesions (like prostate cancer), mixed lesions with both components are also generally present [68,86].

Bone metastasis is a frequent event in breast, prostate, lung, kidney, and thyroid cancers, among others. Clinically, metastases show a strong preference for the axial skeleton (such as the spine, pelvis, and ribs), which is highly vascularized and rich in hematopoietic marrow. This distribution is often attributed to the vertebral-venous (Batson’s) plexus, a low-pressure valveless system that allows venous blood from the breast and pelvis to bypass the lungs and gain access to the spine.

While the axial skeleton is the common target, specific distribution patterns vary by primary tumor type. For breast cancer, common sites include the spine, hips, and skull, with one large study identifying the most frequent sites as the ribs (13.4%), thoracic spine (12.4%), pelvis (12.2%), and lumbar spine (12.1%) [87]. Prostate cancer shows a similar axial pattern, with the most common sites being the spine (32.6%; specifically lumbar 18.3% and thoracic 12.2%), ribs (25.7%), and pelvis (16.1%) [88]. Lung cancer also frequently metastasizes to the ribs (62.3%), thoracic spine (53.8%), and lumbar spine (40.4%) [89]. Renal cell carcinoma commonly metastasizes to the spine (thoracic 39.5%, lumbar 36.8%), followed by the pelvis (26.3%) and ribs (23.7%) [90]. Finally, differentiated thyroid cancer primarily targets the spine (34.6%; specifically thoracic 10.4% and lumbar 8.6%), pelvis (25.5%), and thorax (sternum and ribs, 18.3%) [91].

### 2.4. Experimental Models and Their Limitations

To investigate these complex processes, researchers employ a range of experimental models [92,93,94]. In vitro models allow for controlled study of specific cellular interactions [95]. Recent advances in this area include sophisticated organ-on-a-chip platforms and 3D co-culture systems that can better mimic aspects of the BME [95,96]. Ex vivo models, using cultured bone fragments, provide a native matrix context but lack systemic circulation [61].

In vivo models in animals are essential for studying the complete process [61,97,98,99,100]. The choice of model often depends on the research question, and their success rates can be highly variable [63,100]. Left ventricle injection introduces cells into the arterial circulation, modeling the later stages of dissemination [92]. Intratibial injection places cells directly into the bone marrow to focus on tumor-bone interactions, offering high reproducibility but introducing confounding tissue injuries. The most comprehensive approach is orthotopic injection, where cells are implanted in the primary organ site, allowing for recapitulation of the entire metastatic cascade. However, this model often suffers from high experimental variability and nonsynchronous metastasis, making specific stages difficult to study quantitatively.

A critical limitation across many of these models is that their outcomes do not always mirror clinical patterns [100,101]. While human metastases favor the axial skeleton, many experimental models report a high incidence of lesions in the long bones of the limbs [101]. This discrepancy complicates the preclinical testing of therapies, as drugs may show efficacy in a model that does not accurately reflect the BME of the most clinically relevant metastatic sites [61]. Emerging techniques like spatial transcriptomics are beginning to map these microenvironments at a single-cell resolution, which may help bridge this translational gap [61].

While these models have provided valuable insights into the hematogenous route of metastasis, they may not fully capture alternative dissemination pathways [64,102]. The next section will therefore examine emerging evidence for the lymphatic system’s role in bone metastasis.

## 3. Lymphatic Biology in the Skeletal System

### 3.1. Anatomical Breakthrough: The Discovery of Lymphatic Vessels in Bone

For over a century, the skeletal system was considered an organ devoid of lymphatic vasculature, a view that stemmed from the technical challenges of visualizing these delicate structures within mineralized tissue [103]. This long-held paradigm was fundamentally overturned just recently in a pivotal 2023 study from Biswas et al., which used high-resolution imaging to demonstrate the existence of functional lymphatic vessels in both mouse and human bone [104]. This study revealed that bone lymphatic vessels expand dramatically in response to stress, such as radiation or chemotherapy, a process driven by signaling molecules like IL-6 [104]. Furthermore, these vessels actively support regeneration by secreting lymphangiocrine factors specialized signaling molecules released by lymphatic cells to influence the behavior of other nearby cells like CXCL12, which are critical for the recovery of both bone and hematopoietic stem cells [104]. This anatomical breakthrough provides a credible basis for exploring the direct involvement of the lymphatic system in bone pathology, including metastasis [20,104,105]. This discovery raises the possibility that cancer cells may exploit these intraosseous lymphatic networks not only for drainage but as signaling hubs that influence tumor cell behavior within the bone marrow niche itself.

### 3.2. Immune Trafficking, Homeostasis, and Biomarker Transport

The discovery of these vessels suggests they may function as critical regulators of skeletal immune surveillance and fluid homeostasis [106], a role consistent with lymphatic function in other tissue [107]. While the Biswas et al. study focused primarily on the role of these vessels in regeneration, their discovery is significant as it provides a previously missing anatomical basis for understanding how cellular and molecular traffic, including potential tumor cell dissemination, is regulated within the bone microenvironment [34]. For instance, the presence of this network suggests a potential route for the migration of dendritic cells specialized immune cells that present antigens and for the resolution of local inflammation, which are known functions of lymphatic vessels in other tissues [108,109]. This provides a plausible framework for how the skeletal system, a primary immune organ, manages the egress and trafficking of immune cells [20,110,111,112]. Furthermore, this lymphatic network may serve as a key conduit for various molecular and cellular components from the BME, which could reflect the state of skeletal health or disease [113]. This has significant clinical implications; for instance, the transport of specific disease-associated signatures via intraosseous lymphatics could potentially be developed into novel liquid biopsy approaches for the early diagnosis of skeletal diseases [113,114]. However, direct evidence for this transport route is currently lacking, and it remains a speculative but highly important area for future investigation [113,114,115].

## 4. The Lymphatic System’s Involvement in Skeletal Metastasis

### 4.1. Lymph Node and Lymphatic Invasion and Their Possible Link to Bone Metastasis

Lymphangiogenesis, the formation of new lymphatic vessels, is a hallmark of tumor progression [116,117]. In many cancers, this is not a passive byproduct of inflammation but a process actively promoted by cancer cells. The vascular endothelial growth factor (VEGF)-C and VEGF-D ligands and their receptor VEGFR-3 are the canonical regulators of this process [118]. Notably, in osteotropic malignancies, high VEGF-C expression has been consistently correlated with lymph node metastasis (LNM), particularly in breast [119,120] and prostate cancer [121,122]. Similarly, in melanoma, VEGF-C overexpression is a well-established driver of tumor lymphangiogenesis [123,124] and sentinel lymph node metastasis [125], further supporting the lymphatic route of dissemination [125,126]. Given the robust association between LNM and subsequent bone metastasis (Table 1), this raises the possibility of an indirect mechanistic link. However, direct clinical evidence specifically linking VEGF-C levels to skeletal recurrence as opposed to general distant spread remains limited and requires further investigation.

This correlation is mechanistically plausible given that interfering with this pathway impacts distant metastasis in preclinical models. In mouse models of breast cancer and melanoma, blocking the VEGF-C/VEGFR-3 signaling pathway was effective in inhibiting lymphangiogenesis and reducing regional lymph node metastasis [127,128]. However, the impact on distant organ colonization was variable. While some studies demonstrated that inhibiting this axis significantly reduced metastasis to distant organs, predominantly to the lung [129,130], others reported that the effect was largely confined to regional control without significantly altering the systemic metastatic burden. These findings provide mechanistic evidence in visceral metastasis models that the tumor-induced lymphatic pathway can facilitate systemic spread; however, whether these pathways specifically facilitate osteotropism remains undefined and requires validation using bone-tropic cancer models such as MDA-MB-231-BO breast cancer or PC3-ML prostate cancer cells.

Bone metastasis is a frequent event in breast, prostate, lung, kidney, thyroid cancers and melanoma. The incidence rates and risk associations for these malignancies are synthesized in Table 1. It is important to note that these figures primarily reflect the cumulative incidence in patients with advanced or metastatic disease. The specific anatomical definitions of the primary drainage lymph nodes for these malignancies are summarized in Table 2. As detailed in this table, these basins are not arbitrary surgical landmarks but are validated by anatomical mapping studies such as lymphoscintigraphy and SPECT/CT as the initial biological gateways for dissemination [131,132,133]. Their clinical importance is further underscored by the quantitative data presented in Table 1, where tumor involvement within these specific regions is consistently associated with a significantly increased risk of subsequent bone metastasis. For instance, in breast cancer, involvement of the axillary basin is a major risk factor (OR 2.60) [134], and in prostate cancer, metastasis to the true pelvic nodes serves as the strongest independent predictor of progression to distant bone lesions [50]. Notably, in melanoma, the high hazard ratios (HR) reported in Table 1 underscore that regional lymph node involvement is the strongest independent predictor for subsequent distant dissemination, outpacing other prognostic factors.

### 4.2. The Role of Lymph Node Dissection and Its Impact on Cancer Metastasis

Lymph node dissection (LND) has historically been performed for staging, debulking, and with curative intent. However, the therapeutic benefit of LND in preventing distant metastasis remains a subject of intense debate, as summarized in Table 3. High-level evidence from randomized trials indicates that extensive surgical clearance often fails to improve overall survival in unselected populations. The recently reported SOUND trial in early breast cancer found no significant difference in 5-year distant disease-free survival between sentinel lymph node biopsy (SLNB) and observation (97.7% vs. 98.0%) [149]. Notably, approximately 98% of patients in this trial received breast radiotherapy, and the majority of hormone receptor–positive patients received endocrine therapy, suggesting that modern adjuvant treatments effectively eradicated microscopic nodal disease [149]. Parallel findings were observed in melanoma; the MSLT-II trial demonstrated that completion lymph node dissection (CLND) in sentinel-node-positive patients did not result in a significant survival benefit compared to observation (HR 1.08) [150]. These findings imply that in the era of effective systemic therapy, physical surgical removal may become redundant for survival extension.

However, the therapeutic benefit of LND appears to be context-dependent. While prophylactic removal may fail if systemic dissemination has already occurred, removing established nodal disease can eliminate an active reservoir of disseminating cells [159]. This is best exemplified in prostate cancer. Although a randomized phase 3 trial by Touijer et al. found no significant benefit of extended dissection regarding biochemical recurrence [160], an updated analysis revealed that extended dissection significantly reduced the risk of distant metastasis (HR 0.75, 95% CI 0.64–0.88) (Table 3) [153].This dichotomy reveals a critical insight: removing a metastatic reservoir provides a tangible benefit in blocking the secondary wave of spread. Similarly, in thyroid cancer, LND confers meaningful benefits in locoregional disease control [144,158], establishing distinct clinical roles beyond survival extension. Thus, the value of LND depends on eliminating the metastatic reservoir rather than mere prophylactic staging.

### 4.3. Molecular Drivers of Tumor Lymphangiogenesis and Their Link to Bone Tropism

The transition from lymphatic dissemination to bone metastasis is hypothesized to be orchestrated by specific molecular axes that govern both directionality and survival. Chief among these is the proposed ‘Chemokine Receptor Switch,’ wherein tumor cells sequentially exploit distinct chemokine gradients to navigate from primary site to lymph node and ultimately to bone [161,162]. Mechanistically, VEGF-C/VEGFR-3 signaling induces lymphangiogenesis [163], physically expanding exit routes for cancer cells. However, structural expansion alone is insufficient; the directionality of metastasis is proposed to be governed by sequential chemokine receptor expression, termed the Chemokine Receptor Switch. Initially, tumor cells express CCR7 to migrate toward CCL21-rich lymph nodes [164]. Within the nodal microenvironment, it is hypothesized that cancer cells undergo phenotypic reprogramming, downregulating CCR7 and upregulating CXCR4 [165].

Supporting evidence exists at the endpoints of this proposed trajectory: bulk tissue analyses document CXCR4 upregulation in established bone metastases compared to primary tumors [166], while CCR7 expression has been confirmed in nodal metastases [167]. However, these cross-sectional observations cannot establish causality or temporal sequence. The precise dynamics of the proposed CCR7→CXCR4 transition specifically, whether it occurs during lymph node residence remain undefined. To definitively validate this trajectory, future studies utilizing single-cell RNA sequencing (scRNA-seq) are required to demonstrate the transition from a primary CCR7^high^/CXCR4^low^ phenotype to a nodal CCR7^low^/CXCR4^high^ state [164,168].

To further distinguish between these hypotheses, beyond the phenotypic changes observable by scRNA-seq, clonal tracking studies become essential. This switch is critical because CXCL12, the ligand for CXCR4, is constitutively expressed at high levels in the bone marrow niche [169]. However, alternative models must be considered. Cancer cells may acquire CXCR4 expression stochastically in the primary tumor, with CXCL12 gradients in both lymph nodes and bone marrow independently selecting for pre-existing CXCR4+ clones [170]. Distinguishing between active “education” in the lymph node versus passive selection requires clonal tracking studies.

### 4.4. Remodeling of the Lymphovascular Niche as a Determinant of Bone Metastasis

While the chemokine switch directs cancer cells toward the bone [171], their capacity to survive and colonize the bone marrow is likely acquired through extensive remodeling of the lymphovascular niche [172]. In response to tumor-secreted cytokines and growth factors, the lymph node is actively reshaped into an immunosuppressive state [173,174], characterized by the upregulation of immunosuppressive mediators such as indoleamine 2,3-dioxygenase (IDO) [175], which depletes tryptophan and inhibits T-cell proliferation, and transforming growth factor-β (TGF-β) [176], which promotes regulatory T-cell differentiation and directly induces EMT in tumor cells. This environment is hypothesized to drive metabolic reprogramming toward fatty acid oxidation (FAO) [177]. While FAO dependence has been documented in hematopoietic stem cells within the bone marrow niche [178], whether lymph node-resident tumor cells undergo similar metabolic adaptation remains speculative and requires metabolomic profiling of matched samples to confirm.

A key hypothesis proposes that the lymph node microenvironment initiates a phenotypic shift toward osteomimicry. Current evidence for osteomimicry comes primarily from analysis of established bone metastases, where Runx2 expression is well-documented [179,180]. However, the critical question whether this program is induced within the lymph node or only after arrival in bone remains unanswered. The lymph node priming hypothesis posits that environmental stressors such as relatively low oxygen tension in specific lymph node niches and fluid shear stress within lymphatic sinuses could activate transcription factors upstream of Runx2, such as HIF-1α [181] and mechanosensitive YAP/TAZ [182,183]. Supportive evidence for this mechanism includes observations that hypoxia can modulate Runx2 expression in mesenchymal stem cells, potentially upregulating it under specific conditions in vitro and that lymph nodes and bone marrow share CXCL12-rich niches [168].

Furthermore, phylogenetic studies in a subset of patients have shown that nodal and distant metastases, including bone lesions, can be more closely related to each other than to the primary tumor, although in most cases distant metastases appear to arise directly from the primary tumor [184]. This suggests that while not the exclusive route, the remodeled lymph node may serve as a pivotal intermediate stage where cancer cells acquire osteotropic traits before systemic dissemination (Figure 1B). However, direct demonstration of Runx2 induction within lymph nodes is lacking. To bridge this gap, critical experiments are required, including immunohistochemistry comparing Runx2 expression across matched primary, nodal, and bone samples, in vivo lineage tracing using Runx2-reporter mice, and scRNA-seq trajectory analysis to identify the precise anatomical site of the osteomimetic transition. Consequently, determining the magnitude of this lymphatic contribution relative to direct hematogenous spread remains a key priority for future research.

## 5. The Lymphatic–Bone Axis: Hypothesized Mechanisms of the Lymphatic–Bone Axis and Future Perspectives

### 5.1. Experimental Evidence for the Lymph Node as a Gateway for Systemic Dissemination

Recent studies utilizing distinct methodological approaches have provided experimental evidence in mouse models suggesting that the lymph node can serve as a physical gateway for systemic dissemination [185,186]. Pereira et al. employed a photoconvertible lineage-tracing strategy using Dendra2 in melanoma and breast cancer xenografts [186]. This non-invasive approach allowed for the longitudinal tracking of naturally emigrating cancer cells, confirming that specific clones exited the node to appear as circulating tumor cells (CTCs) and subsequently seeded distant metastases. Complementing this, Brown et al. used a microinfusion model to introduce tumor cells directly into afferent lymphatics. While more artificial, this method captured the early kinetics of dissemination, revealing that tumor cells could invade high endothelial venules (HEVs) and enter the venous circulation as early as three days post-infusion. This distinction is pivotal because Pereira’s work confirms the natural occurrence of this trajectory over time, whereas Brown’s work highlights the rapidity with which nodal tumor cells can gain access to the bloodstream, providing a more direct route to the systemic circulation compared to passive drainage through the thoracic duct.

These functional assays in animal models are further supported by retrospective phylogenetic analysis in humans. Naxerova et al. reconstructed the evolutionary lineage of metastatic lesions in colorectal cancer patients, demonstrating that in approximately 35% of cases, distant metastases shared a more recent common ancestor with lymph node metastases than with the primary tumor [184]. Although this study focused on colorectal cancer, the fundamental principle of the lymph node as a reseeding hub may apply to osteotropic malignancies like breast and prostate cancer, though differences in metastatic biology warrant similar phylogenetic reconstruction in these specific contexts to confirm generalizability.

However, extrapolating these findings to the lymphatic–bone axis requires careful scrutiny. First, the mouse models used, such as 4T1 [187,188] and B16F10 [189,190], exhibit an intrinsic organotropism toward the lung, leaving the step from systemic circulation to bone colonization unverified in these systems. Second, fundamental questions remain regarding the human relevance of this pathway, specifically what proportion of bone metastases actually utilize this route versus direct hematogenous spread in patients. Furthermore, it remains to be determined whether the lymph node microenvironment is truly instructive, conferring osteotropic traits, or merely permissive, allowing pre-existing aggressive clones to pass through. Importantly, direct in vivo evidence demonstrating that circulating tumor cells derived specifically from lymph nodes possess a higher bone colonization efficiency compared to those from the primary tumor is currently lacking. Addressing these uncertainties requires the comprehensive experimental approaches outlined in the following section.

### 5.2. Therapeutic Approaches for Targeting the LNM-BM Axis

While these mechanistic insights remain incomplete, the clinical urgency of bone metastasis prevention necessitates the parallel development of therapeutic strategies, even as our understanding of the LNM-BM axis continues to evolve. The translation of biological concepts into clinical benefit has proven challenging, as evidenced by major adjuvant trials such as AZURE (zoledronic acid) [191,192] and D-CARE (denosumab) [193], which failed to improve bone metastasis-free survival in the overall population. This consistent failure likely reflects multiple converging factors. First, from a timing perspective, interventions may have been administered after systemic dissemination had already occurred [194]. Second, from a mechanistic standpoint, targeting the bone microenvironment alone is likely insufficient if the seed has already acquired osteotropic traits in the lymph node [193]. Third, from a trial design perspective, the lack of biomarker-driven enrichment diluted potential benefits in unselected populations [191].

Consequently, should the lymphatic priming hypothesis be definitively validated, a paradigm shift toward dual-targeting strategies would be warranted. Conceptually attractive approaches involve combining agents that block lymphatic transit, such as VEGFR-3 inhibitors, with those that modify the bone niche [195,196]. However, a major hurdle is that DTCs in the bone marrow often enter a prolonged quiescent state [197], which may persist for years or decades. Current bone-modifying agents primarily target active osteolysis and may have limited effect on dormant DTCs [198]. Understanding whether lymph node priming influences the molecular determinants of dormancy entry including p27 [199], NR2F1 expression [200] or the susceptibility to reactivation cues, notably inflammatory cytokines, osteoclastic activity [201] is therefore a priority that could inform the timing and selection of adjuvant interventions.

Finally, future trials must move beyond unselected cohorts by employing biomarker-driven strategies. Potential enrichment criteria include high VEGF-C expression in the primary tumor or radiographic evidence of lymphangiogenesis detectable by contrast-enhanced MRI. Genomic signatures associated with both lymphotropism and osteotropism, such as the co-expression of specific adhesion molecules and stemness-associated markers (e.g., the broadly expressed hyaluronic acid receptor CD44 [202] and ALDH1 [203]) and bone-homing receptors (CXCR4 [165,168], integrin αvβ3 [105]), could provide additional stratification. While differentiating lymph node-derived CTCs from primary-derived CTCs remains a technological hurdle, emerging approaches such as single-cell DNA barcoding [204], multi-region methylation profiling [205], or mitochondrial DNA heterogeneity analysis [206] may enable the retrospective tracing of CTC origin, potentially addressing this gap within the next 3–5 years, though clinical validation will require additional time.

## 6. Conclusions

Bone metastasis remains a major determinant of morbidity and mortality in osteo-tropic cancers; however, a strictly hematogenous model does not adequately explain the strong and reproducible clinical association between lymph node involvement and subsequent skeletal dissemination. The Lymphatic–Bone Axis proposed in this review re-frames lymph nodes as biologically active intermediates rather than passive conduits, emphasizing their capacity to shape metastatic competence. Available evidence suggests that the lymph node microenvironment can promote phenotypic adaptations in tumor cells, including remodeling of chemokine receptor programs, acquisition of bone-mimetic and stress-adaptive traits, and metabolic changes that may enhance survival within the bone marrow niche. In parallel, experimental observations of direct tumor cell entry into the bloodstream via intranodal high endothelial venules provide an anatomical explanation for why surgical nodal clearance alone often fails to prevent distant relapse.

Important questions nevertheless remain unresolved. It is still unclear whether lymph nodes actively instruct osteotropism or preferentially select for pre-existing bone-competent subclones, and the quantitative contribution of this pathway to human bone metastasis relative to direct hematogenous spread is unknown. Resolving these questions will require longitudinal analyses of matched primary tumors, lymph nodes, and bone lesions, integrated with single-cell and spatial approaches and functional comparisons of lymph node-derived tumor cells. Such mechanistic resolution has direct translational implications, suggesting that interventions focused solely on the bone microenvironment may be insufficient once tumor cells have undergone lymphatic conditioning. Therefore, integrating strategies that disrupt lymphatic transit or nodal priming with bone-targeted therapies may represent a more effective framework for preventing, rather than simply managing, skeletal metastasis.

## Figures and Tables

**Table 1:** Incidence of bone metastasis and quantitative association of lymph node metastasis (LNM) and lymphovascular invasion (LVI) with skeletal risk.

Cancer Type	Incidence of Bone Metastasis	Risk Factor	Association with Bone/Distant Metastasis	Reference
Breast cancer	65–75% (Advanced Stage)	LNM	OR 2.60 (95% CI 1.41–4.80)—Independent predictor of BM	[47,48]
		LVI	HR 2.37 (*p* = 0.001)—Independently increases risk of distant metastasis	[48,49]
Prostate cancer	65–90% (Advanced Stage)	LNM	OR 7.15 (95% CI 1.54–33.24)—Strong association with BM presence	[50]
		LVI	HR 2.2 (95% CI 1.6–3.0)—Independent predictor of metastasis	[51]
Lung cancer	30–40% (NSCLC)	LNM	Significant correlation with skeletal events (*p* < 0.05; Effect size not reported)	[52,53]
		LVI	HR 1.73 (95% CI 1.24–2.41)—Increases risk of overall recurrence/mortality (not bone-specific)	[54]
Kidney cancer	20–25% (Advanced Stage)	LNM	OR 2.45 (95% CI 1.98–3.03)—Predicts synchronous BM	[55]
		LVI	HR 4.0 (*p* = 0.026)—Independent predictor of DFS even in organ-confined disease	[56]
Thyroid cancer	2–13% (Differentiated Thyroid Cancer)	LNM	OR 1.86 (*p* < 0.001)—Increases risk of developing BM	[57]
		LVI	Highly prevalent (92.6%) in BM cases; reflects prevalence rather than predictive risk	[58]
Melanoma	17–43% (Advanced Stage)	LNM	HR 6.53 (95% CI 3.39–12.58)—Strongest independent prognostic factor for survival	[59]
		LVI	HR 5.4 (95% CI 1.6–18.4)—Independent prognostic factor for overall survival	[60]

Abbreviations used in this table include BM (Bone Metastasis), LNM (Lymph Node Metastasis), LVI (Lymphovascular Invasion), OR (Odds Ratio), HR (Hazard Ratio), CI (Confidence Interval), DFS (Disease-Free Survival), NSCLC (Non-Small Cell Lung Cancer), and DTC (Differentiated Thyroid Cancer).

**Table 2:** Anatomical definitions of primary lymphatic drainage basins in major osteotropic cancers.

Cancer Type	Primary Drainage Lymph Nodes	Specific Node Groups	Evidence of Drainage Pattern (Mapping/Anatomical Study)	Reference (Anatomy)
Breast cancer	Axillary LNs, Internal Mammary LNs, Supraclavicular LNs	Level I–III Axillary Nodes, Internal Mammary Chain, Supraclavicular Nodes	Lymphoscintigraphy in >500 patients confirmed the axilla as the dominant sentinel basin, with concurrent drainage to internal mammary nodes observed in approximately 20% of cases.	[135,136,137]
Prostate cancer	True Pelvic LNs (distal to common iliac bifurcation)	Obturator (sentinel), Internal Iliac, External Iliac, Presacral Nodes	SPECT/CT and sentinel node mapping identify the obturator (33.5%), external iliac (32.5%), and internal iliac (22.5%) chains as the most frequent primary landing sites, confirming drainage extends beyond limited boundaries.	[131,132,133]
Lung cancer	Mediastinal & Hilar LNs	Hilar (N1), Mediastinal (N2), Supraclavicular (N3) Nodes	Lobar-specific mapping establishes typical pathways (upper lobes → paratracheal; lower lobes → subcarinal), yet direct mediastinal drainage (skip metastasis) bypassing hilar nodes occurs in up to 25% of cases.	[138,139,140,141]
Kidney cancer	Retroperitoneal LNs	Renal Hilar, Paracaval (right), Para-aortic (left) Nodes	Anatomical mapping reveals laterality-dependent pathways: right-sided tumors drain primarily to paracaval and interaortocaval nodes, while left-sided tumors drain to para-aortic nodes.	[142,143]
Thyroid cancer	Cervical & Upper Mediastinal LNs	Central (Level VI/VII) and Lateral Compartments (Level I–V)	Pattern-of-spread analyses demonstrate that lymphatic spread typically follows a sequential progression from the central compartment to the lateral neck, although ‘skip’ lateral metastases occur in a subset of patients.	[144,145]
Melanoma	Cervical LNs, Axillary LNs, Inguinal LNs (site-dependent regional basins)	Cervical levels II–V and supraclavicular nodes, level I–III axillary nodes, superficial and deep inguinal/femoral nodes, with occasional interval and nonclassical basins demonstrated on lymphoscintigraphy.	Dynamic lymphoscintigraphy shows that while most melanomas drain to expected cervical, axillary, or inguinal basins, truncal and head-and-neck lesions often have multiple or unexpected sentinel nodes, justifying routine preoperative mapping.	[146,147,148]

Abbreviations used include LNs (Lymph Nodes), SPECT/CT (Single Photon Emission Computed Tomography/Computed Tomography), RCC (Renal Cell Carcinoma), and NSCLC (Non-Small Cell Lung Cancer).

**Table 3:** Therapeutic impact of lymph node dissection on distant metastasis and survival outcomes.

Cancer Type	Comparison (Intervention vs. Control)	Cohort Size (*n*)	Result (HR/OR/IRR [95% CI])	Study Design (Reference)
Breast cancer	ALND vs. SLNB	*n* = 3585	OR 0.88 [0.65–1.19] (No survival benefit)	Meta-analysis [151]
	SLNB (Biopsy) vs. No Axillary Surgery (SOUND Trial)	*n* = 1463	No significant difference (5-yr DDFS: 97.7% vs. 98.0%)	RCT [149]
Prostate cancer	PLND vs. No PLND (Propensity Matched)	*n* = 80,287	No significant difference (*p* > 0.05 for all GG)	Cohort [152]
	ePLND vs. limited PLND	*n* = 1432	HR 0.75 [0.64–0.88] (Reduced risk of Distant Metastasis)	RCT [153]
Lung cancer	MLND vs. MLNS	*n* = 1791	HR 0.77 [0.55–1.08] (Trend toward benefit but not significant)	Meta-analysis [154]
	Systematic LND vs. Selective LND	*n* = 1440	HR 1.05 [0.82–1.34] (No survival benefit)	Meta-analysis [155]
Kidney cancer	Radical nephrectomy + LND vs. Radical nephrectomy without LND	*n* = 29,044 (LND) *n* = 106,471 (non-LND)	HR 1.10 [0.95–1.27] (No significant OS benefit)	Meta-analysis [156]
	Cytoreductive nephrectomy + LND vs. Cytoreductive nephrectomy without LND	*n* = 4690 (SEER database)	CSS HR 1.18 [1.09–1.27] OS HR 1.13 [1.05–1.21] (LND associated with worse survival likely due to selection bias)	Cohort [157]
Thyroid cancer	No pCCND vs. pCCND (cN0 PTC)	*n* = 2902	HR 13.362 [2.928–60.986] (Omission increases recurrence risk)	Meta-analysis [158]
	pCCND vs. No pCCND (cN0 PTC)	*n* = 3331	IRR 0.65 [0.48–0.86] (LND reduces recurrence risk)	Meta-analysis [145]
Melanoma	CLND vs. Observation (in SLN-positive patients)	*n* = 1934	HR 1.08 [0.88–1.33] (No significant survival benefit)	RCT [150]

Abbreviations include ALND (Axillary Lymph Node Dissection), SLNB (Sentinel Lymph Node Biopsy), PLND (Pelvic Lymph Node Dissection), pCCND (Prophylactic Central Compartment Neck Dissection), HR (Hazard Ratio), and IRR (Incidence Rate Ratio).

## Data Availability

No new data were created or analyzed in this study. Data sharing is not applicable to this article.
